# Stereoelectroencephalography-Guided Radiofrequency Thermocoagulation: Diagnostic and Therapeutic Implications

**DOI:** 10.3390/brainsci14020110

**Published:** 2024-01-23

**Authors:** James F. Castellano, Shobhit Singla, Niravkumar Barot, Joshua P. Aronson

**Affiliations:** 1 Department of Neurology, University of Pittsburgh School of Medicine, Pittsburgh, PA 15213, USA; singlas@upmc.edu; 2 Department of Neurology, Beth Israel Deaconess Medical Center, Boston, MA 02215, USA; 3 Department of Neurosurgery, Beth Israel Deaconess Medical Center, Boston, MA 02215, USA; jaronson@bidmc.harvard.edu

**Keywords:** epilepsy, drug-resistant epilepsy, radiofrequency thermocoagulation, stereoelectroencephalography

## Abstract

Despite recent medical therapeutic advances, approximately one third of patients do not attain seizure freedom with medications. This drug-resistant epilepsy population suffers from heightened morbidity and mortality. In appropriate patients, resective epilepsy surgery is far superior to continued medical therapy. Despite this efficacy, there remain drawbacks to traditional epilepsy surgery, such as the morbidity of open neurosurgical procedures as well as neuropsychological adverse effects. SEEG-guided Radiofrequency Thermocoagulation (SgRFTC) is a minimally invasive, electrophysiology-guided intervention with both diagnostic and therapeutic implications for drug-resistant epilepsy that offers a convenient adjunct or alternative to ablative and resective approaches. We review the international experience with this procedure, including methodologies, diagnostic benefit, therapeutic benefit, and safety considerations. We propose a framework in which SgRFTC may be incorporated into intracranial EEG evaluations alongside passive recording. Lastly, we discuss the potential role of SgRFTC in both delineating and reorganizing epilepsy networks.

## 1. Introduction

Epilepsy is estimated to affect 50–70 million people worldwide, with anti-seizure medications remaining the mainstay of treatment. Despite continued development of novel medications, an estimated 30–40% of patients with seizures develop drug-resistant epilepsy (DRE) [1,2,3]. DRE, as defined by the International League Against Epilepsy, is the “failure of adequate trials of two tolerated, appropriately chosen and used antiepileptic drug schedules (whether as monotherapies or in combination) to achieve sustained seizure freedom” [4]. Critical for patient quality of life, the negative impact of seizures on neuropsychological function has been well documented, as has the risk of sudden death in epilepsy (SUDEP) [5,6]. There is also a large economic burden. In the United States, with approximately 2.3 million epilepsy patients, estimates of annual direct per-person healthcare costs range from USD 1022 to 19,749 (in 2013 USD), but analysis by subgroup reveals that for patients with DRE, costs are more than double the costs of well-controlled, stable patients [7].

For patients with DRE, the morbidity and mortality of uncontrolled seizures often lead to referral for possible surgical interventions. Epilepsy surgery continues to be guided by an understanding of the seizure onset region and the seizure network; current treatment algorithms rely on the confidence of that localization [8]. Identification of these regions routinely requires scalp EEG investigations in epilepsy monitoring units (EMU), so called Phase 1 evaluations. Often in more complex and non-lesional cases, invasive neuromonitoring is performed within an EMU in a Phase 2 evaluation. Stereoelectroencephalography (SEEG), intracerebral EEG recording from electrodes stereotactically placed through small holes in the skull, has largely supplanted subdural electrode monitoring due to superior patient acceptability and decreased complication risk [9]. After identification of relevant epileptogenic sites, treatment options include open resection, minimally invasive ablation, and neuromodulation approaches.

Traditional resective surgery has been demonstrated to be superior to medical therapy for seizure control in DRE [10,11] and is associated with neuropsychological improvement [12,13]. However, there are significant surgical and neurological risks associated with resection [14]. Efforts to minimize resection through keyhole and selective approaches are technically challenging, and multiple studies report conflicting outcomes for seizure freedom [15,16,17,18]. This has spurred a drive towards minimally invasive ablative approaches. Magnetic resonance-guiding laser interstitial thermal therapy (MRgLITT) can specifically target the mesial temporal structures or other well-defined regions via a 2.2-mm twist drill craniotomy and laser fiber. This technique is limited to a radius of approximately 8-mm around the fiber with the length of the ablation controlled by a creating a series of ablations along a linear trajectory. Reported seizure freedom rates have varied, but a recent large series reported a 58% Engel I outcome at 2 years post-ablation with a complication rate of 1.3% for radiographic hemorrhage and 0.4% rate of permanent neurologic deficit from the hemorrhage [19]. Studies of long-term neuropsychological results are limited, but small series have shown naming and facial recognition deficits are lower for selective laser amygdalohippocampotomy versus open temporal lobectomy [20]. For patients who are not candidates for resection or ablation due to multifocal epilepsy or seizure onset within or abutting the eloquent cortex, neuromodulation approaches with deep brain stimulation and responsive neurostimulation are often employed. However, there are several reports in multifocal epilepsy where resection or ablation of safe regions is combined with neuromodulation in a multimodal treatment [21,22].

Surgical resection, laser ablation, and neuromodulation are additional surgical procedures beyond the SEEG evaluation. However, SEEG electrodes that have been found to be within the seizure onset zone may be utilized as an alternative to staged interventions. Radiofrequency thermocoagulation (RFTC) involves the passage of current via a radiofrequency generator, thereby generating focal thermal lesions [23]. This approach can be performed during Phase 2 intracranial recording evaluation. While radiofrequency lesions date back to the 1960s, early efforts using RFTC were discouraging, as the lesions were relatively small and targeted based on anatomy [24]. The innovation of SEEG-guided radiofrequency thermocoagulation (SgRFTC) is the incorporation of ictal and interictal recordings to guide the precise contacts used for ablation. The SEEG electrode remains in place, ensuring precise localization of tissue destruction. This may improve the efficacy of the technique, as the goal is not the ablation of an anatomical location but an electrographic seizure onset zone and/or other relevant nodes in the epileptic network. There are several advantages to SgRFTC that are well delineated by Bourdillon, et al.: (1) the SEEG recordings and stimulation mapping can guide targeting, (2) multiple lesions may be created between adjacent contacts, and (3) the SEEG electrodes are already implanted, so no additional surgical or anesthesia risks are introduced [25]. There is increasing interest in SgRFTC and the US Food and Drug Administration recently cleared the first system designed for SgRFTC that allows for precise temperature control within the ablation (FDA 5109k K231675). Thus, the use of SgRFTC will likely expand in the coming years. This review outlines the historical experience of SgRFTC as a therapeutic intervention and proposes how it may be incorporated in a modern algorithm for the management of patients with DRE.

## 2. International Experience with SgRFTC

Radiofrequency thermocoagulation is not a new concept in the treatment of epilepsy; it was initially described by Schwab et al. [26] in 1965 with amygdala lesions and later extended to hippocampal lesioning by Flanigin and Nashold in 1976 [27]. Early use of radiofrequency ablative techniques evaluated the methodology as a separate alternative surgical procedure to traditional open resective surgery, specifically anterior temporal lobectomy. Before the widespread utilization of SEEG methodologies, Parrent and Blume [28], in 1999, reported outcomes of 19 patients who underwent stereotactic radiofrequency ablations of the amygdala and anterior hippocampus after completing the standard Phase 1 presurgical evaluation. This cohort was also considered appropriate for anterior temporal lobectomy, and all but one patient had a concordant MRI lesion. Two separate lesioning strategies were used; group one utilized discrete lesions and group two utilized a larger number of confluent lesions. A larger percentage of patients in group two had a favorable seizure outcome (60% vs. 20%), including the only two patients who remained seizure-free at the 9-month follow-up.

Increasing usage of Phase 2 intracranial monitoring with stereotactically-placed depth electrodes has afforded greater opportunities to evaluate this technique across many different epilepsies. Utilizing SEEG electrodes, Bourdillon et al. [29] showed that using unfixed parameters of radiofrequency current delivery on contiguous electrodes (bipolar stimulation) and increasing it until the power delivered by the generator collapsed, produced reproducible lesions. Specifically, in an in vitro setting (egg white) they found that when unfixed radiofrequency parameters were used, there was no increase in lesion volume after the delivered power spontaneously fell down; mean power required to reach this point was 9.6 W, mean voltage was 54.2 V, and mean intensity was 176.9 mA producing a mean lesion volume of 32.9 mm^3^. Importantly, temperature measured in these experiments by means of external monitoring ranged from 78 to 82 °C. In the animal (rabbit) model setting, the researchers found that utilizing fixed stimulations ranging from 5 W for 30 s to 10 W for 90 s, they were able to produce histopathologically similar lesions with increased lesion size in the higher power groups without significant difference in lesion size based on time that the impulse was delivered. Also, the electrodes were not physically damaged in any stimulation setting. From these experiments, they concluded that the most appropriate lesioning strategy was the use of unfixed parameters—increase the power delivered until the intensity and voltage spontaneously fall down—on contiguous contacts. Very importantly, they also conclude that utilizing the aforementioned settings, it is impossible to unintentionally create a lesion of excessive size as the thermocoagulation causes a proportional rise of tissular impedance simultaneously. Later in vitro and in vivo investigations by Staudt et al. [30] importantly illustrated that lower energy over longer durations, as well as increased linear separation of electrodes, resulted in the largest lesion sizes, up to 80 mm^2^.

At University Hospital in Lyon, France, Guénot and colleagues [23] reported a group of 20 patients who underwent standard Phase 2 monitoring using SEEG. Utilizing these SEEG electrodes, lesions were made in contacts that showed either low voltage fast activity or spike wave discharges at seizure onset. No interictal activity was noted after lesioning. Three patients were seizure-free and an additional eight showed >80% reduction in seizure frequency. These patients included those with mesial temporal, lateral temporal, or frontal epilepsies, showing the efficacy of SgRFTC across epilepsy types. In 2017, their group published their 10-year experience with SgRFTC, which included a total of 162 patients. They found that 25% of patients were seizure-free, while 67% had had >50% improvement of their epilepsy (Table 1) [31].

Multiple retrospective studies on factors predictive of a positive response after SgRFTC had various outcomes. A retrospective study of 89 patients done in Italy, including those that were deemed not suitable for resective surgery, found that nodular heterotopia as etiology, patient age, and number of intralesional sites were factors that correlated with outcome [32]; another study found only presence of an MRI lesion as a significant predictor of favorable outcome [33]. Other factors including extent of interictal discharges, location of seizure onset zone, and ratio of coagulated sites showed no statistical significance [33].

In addition to the European experience, there has been extensive utilization of this procedure at the Montreal Neurologic Institute [34]. Mirza et al. [34] performed radiofrequency thermocoagulation on 14 patients with drug-resistant epilepsy. Like the Lyon group, they utilized axial coagulation (between contiguous contacts on a single electrode). Additionally, they also utilized cross coagulation between electrodes within a 5 mm distance. Like others, they found SgRFTC most effective in MRI lesional cases, specifically unilateral PVNH and focal cortical dysplasia; 2 in 3 patients with PVNH were seizure-free at one year and 1 in 4 patients with focal cortical dysplasia achieved Engel 1 outcomes (Table 1). Regarding adverse events, the Montreal group noted transient post-operative complications in 2 of 14 patients: one with a verbal memory deficit with spontaneous resolution and the other with contralateral weakness due to SMA region edema, which responded to steroid administration (Table 2). Additionally, they noted occasional transient vague headache or facial pain in the trigeminal distribution reported by patients during the procedure, which was attributed to proximity of the electrode to the tentorium of Meckel’s cave region.

Given the heterogeneity found in different studies (Table 1), a larger meta-analysis published in 2018 reported outcomes on 296 patients across six retrospective studies and found that 23% of pooled patients (CI—8–50%) achieved seizure freedom at one year [35]. Among these studies, a consistent trend was noted with better response in patients with nodular heterotopia, who had the highest proportion of responder rate (81%), while those with non-lesional MRIs had the least (41%). However, these results were not statistically significant [35]. Among these studies, the seizure freedom rate ranged from 7% to 71%, likely due to variability in etiologies and target selection, as some centers ablated areas with electrodes showing a spike/wave pattern or LVFA (low voltage fast activities), while others targeted a larger cortical volume with the aim of destroying the larger part of the epileptogenic zone. Regarding safety, importantly, only five patients (2.5%) had permanent neurological deficits following the procedure (Table 2). In four of these patients, a neurological deficit was expected since lesions were made in the primary motor cortex. A recent single-center study, which also included patients with various etiologies, reported a similar efficacy as the above metanalysis with 12 out of 49 patients (24.5%) having an Engel 1 outcome after SgRFTC [36]. Notably, this group also reported that 15 out of 32 patients who did not achieve Engel 1 after SgRFTC alone went on to later achieve a class 1 outcome with a secondary procedure (laser ablation or open resection), suggesting that SgRFTC fails when the epileptogenic zone is larger than the SgRFTC-lesioned volume and may be more effective in patients with smaller and well localized EZ [36]. Furthermore, they also reported a longer transient time of seizure freedom after SgRFTC as a predictor of outcome after the second procedure [36].

Table 1 Retrospectively reported epilepsy outcomes after SGRFTC in cohorts *n* > 5 between 2013 and 2022. Responder defined as >50% seizure reduction. Asterisk indicates that responders received additional surgical interventions after SgRFTC.

Table 2 Permanent and transient neurologic deficits from 11 retrospective series (428 patients). Expected deficits defined as neurologic deficits anticipated by performing physicians and directly relatable to the localization of radiofrequency lesion.

One of the recent studies that mainly focused on patients with hypothalamic hamartoma (HH) showed a remarkable seizure freedom rate, with 23 out of 28 patients achieving Engel Class 1 outcome with at least 12 months follow-up [37]. Of note, this rate, albeit from a single study, is better than the previously reported retrospective efficacy of MRgLITT in hypothalamic hamartoma [38]. Another study by Fan et al. [39] showed that when patients with mTLE with clear hippocampal sclerosis were treated with SgRFTC, it resulted in 76% of patients achieving an Engle Class I outcome. Similar favorable results (4 out of 5 patients) in patients with mTLE seizure-free at 12 months follow-up were replicated, however, in a smaller cohort by Losarcos et al. [40]. Again, these rates, albeit from smaller cohorts, are superior to MRgLITT [38]. These three studies further emphasize that as a therapeutic strategy, SgRFTC is best suited for patients with smaller and well-localized epileptogenic zones. Overall, although our current experience is limited to retrospective studies, SgRFTC appears to be a safe procedure (Table 2) with excellent efficacy in correctly chosen populations.

## 3. Future of SgRFTC

We have reviewed the international experience with SgRFTC as a minimally invasive epilepsy surgery technique. In this section, we discuss the future of SgRFTC as both a therapeutic intervention and a diagnostic technique. The surgical evaluation of pharmacoresistant epilepsy is often highly individualized, utilizing multi-modal data to best ensure a successful outcome, traditionally defined as seizure freedom. We propose that prudent use of SgRFTC may not only definitively treat drug-resistant epilepsies but also provide novel diagnostic information that may better inform alternative surgical approaches.

Regarding treatment strategies, the classic paradigm has been precise destruction of the epileptogenic zone by passing current between contiguous contacts on a single electrode, thereby creating a thermocoagulative lesion. Prior systematic reviews and meta-analyses [41] have illustrated the efficacy of SgRFTC in focal, anatomically-limited epilepsies. Particular etiologic examples include hypothalamic hamartomas [42,43,44,45,46], hippocampal sclerosis [39], focal cortical dysplasias [34,47,48,49], and periventricular heterotopias [35,42,50]. While current reported treatment outcomes are quite good, reaching up to 60% Engel 1 status [41], novel methodologies and applications may not only improve seizure freedom rates but also widen the scope of treatment.

As mentioned, most methodologies involve utilization of contiguous contacts on a single electrode, or two-dimensional planning. Newer methods extend the technique to three-dimensional geometry by passing current both along contiguous contacts of a single electrode and between contacts on different but proximate electrodes. Several reports have illustrated the benefits of the technique, namely larger lesion sizes [51,52]. Using a combination of orthogonal and oblique sEEG implant approaches, structures such as the mesial temporal structures [53] and insular cortices [52,54] may be particularly amenable to this approach. While 3D lesioning may increase RF lesion sizes to something more akin to MRgLITT (albeit without the need for a second procedure), the use of small precise lesioning afforded by SgRFTC may also be of great value in certain clinical conditions. Losarcos et al. [40] have recently proposed the use of SgRFTC to precisely lesion the dominant mesial temporal structures to disrupt circuitry between the amygdala and hippocampus but preserve neocortical connections, much akin to the open surgical technique of multiple hippocampal transections. In their small cohort, they reported that 4 of 5 patients were seizure-free at 14–18 months of follow-up and no patients had subjective memory complaints at 6 months. Just as the accurate lesioning of SgRFTC may be utilized to target fiber tracts, it may also be advantageous for sparing functional tracts and thereby limiting neurological morbidity; Song et al. [55] have recently illustrated the utilization of diffusion spectrum imaging tractography to complement SgRFTC in optic radiation-sparing interventions. Another opportunity for precise lesioning by SgRFTC builds on current evidence for the role for neuromodulation of thalamic structures [56,57] in the generalized epilepsies. Preliminary data from Aguado-Carrillo et al. [58] have suggested a palliative role of radiofrequency ablation of the centromedian thalamic nucleus.

Given the minimally invasive nature and the ability to observe electrophysiological consequences in real time, SgRFTC has unique diagnostic benefits compared to other surgical approaches. Thus, we suggest that SgRFTC and subsequent recording may act as an intermediate step between passive intracranial recording and staged surgical intervention. We propose the term “Phase 2.5” for intracranial recording during and after SgRFTC. Changes in interictal epileptiform abnormalities as well as seizure onset and propagation patterns after SgRFTC may give insights into epileptic network organization. Simula et al. [59] found acute changes in brain network and local activity after SgRFTC, and these changes differed among patients with significant seizure reduction versus those without. Xu et al. [60] have proposed that abolishment of high frequency oscillations by SgRFTC may be a key to success. Furthermore, post-SgRFTC seizure frequency changes may predict efficacy of more conventional surgical procedures; Shields et al. [36] found that longer transient time of seizure freedom after SgRFTC predicted secondary surgery outcomes.

Lastly, SgRFTC provides a particular opportunity to both probe and treat epilepsy networks. Epilepsies, even focal symptomatic, are currently considered network diseases [61,62,63]. Insufficient pre-surgical comprehension of focal epilepsy networks is believed to be a main contributor to poor post-operative outcomes. Given the ability to create multiple lesions across anatomically distinct and often distant locations, SgRFTC of relevant epileptic nodes would allow epileptologists to not only interrogate epilepsy networks but also treat them. Both canonical nodes such as functionally connected cortices and non-canonical subcortical nodes such as thalamic nuclei [64] may be evaluated in this manner. Furthermore, small, precise lesions in epileptic nodes may minimize neurologic deficits as compared to larger resective surgeries. Several groups [65,66] have begun utilizing network ablation strategies with some success. Improvements in intracranial recording evaluation will likely further benefit this strategy; epileptogenic zone fingerprint analysis [67], epileptogenicity ranking methods [68], and advanced computational measures [69] are examples of new methodologies for discriminating epileptogenic tissue.

There is an urgent need for prospective evaluation of SgRFTC methodologies. In the last decade, there have been several novel minimally invasive surgical approaches including LITT [70,71] and SRS [72,73]. Current data are variable regarding both efficacy in terms of seizure freedom and adverse effects, specifically neuropsychological deficits. The fundamental difference between SgRFTC and the other minimally invasive procedures is the inextricable link between RFT and the neurophysiological methods which form the basis of epilepsy localization. Unlike the other techniques, which are principally structurally guided, RFT is functionally guided by intracranial sEEG recordings. When utilized in conjunction with careful SEEG implantation and analysis, RFT allows for direct lesioning of seizure onset zone. Currently, there are two active prospective clinical trials evaluating efficacy of SgRFTC; the first comparing SgRFTC to anterior temporal lobectomy in mesial temporal lobe epilepsy [74] and the second comparing SgRFTC to both MRgLITT and open resection across various focal epilepsies [75]. These prospective efforts, coupled with increased routine clinical utilization of SgRFTC, will not only further inform safety and efficacy but also elucidate novel applications.

In conclusion, we propose SgRFTC as a pivotal tool in both the diagnostic and therapeutic armamentariums of epileptologists and neurosurgeons. The worldwide burgeoning use of SEEG methodologies provides an ideal background for widespread implementation.

## Figures and Tables

**Table 1:** Reported SgRFTC Outcomes.

First Author	Study Date	Sample Size	No. of Patients with Engel Class I at 1 Year	No. of Responders at 1 Year
Wu	2013	7	3	6
Cossu	2015	89	10	17
Bourdillon	2017	162	11	89
Dimova	2017	23	1	8
Mirandola	2017	17	12	14
Zaho	2017	12	5	10
Fan	2019	22	16	4
Mirza	2020	14	3	8 *
Wang	2021	28	23	3
Losarcos	2021	5	4	0
Shields	2022	49	12	NA

**Table 2:** Reported SgRFTC Adverse Event Data.

First Author	Year	Sample Size	Permanent Neurologic Deficit	Transient Neurologic Deficit
Wu	2013	7	0	0
Cossu	2015	89	1 Expected—Right Hemiparesis	1 Expected—Left Foot Motor Deficit
			1 Unexpected: Neuropsychological Syndrome	0 Unexpected
Bourdillon	2017	162	2 Expected—Hand Palsy (1), Ankle Palsy (1)	1 Expected: Brachiofacial Pasly (1)
			0 Unexpected	3 Unexpected: Gritty oral sensation (1), Hemiparesis (1), Partial Aphasia (1)
Dimova	2017	23	1 Expected: Right Thumb Hypoesthesia	2 Expected: Partial hand paresis, Dysgeusia
			0 Unexpected	0 Unexpected
Mirandola	2017	17	0	0
Zaho	2017	12	0	0
Fan	2019	22	0	0
Mirza	2020	14	0	1 Expected: SMA Syndrome
				1 Unexpected: Verbal Memory Deficits
Wang	2021	28	0 Expected	0
			2 Unexpected: Short Term Memory Deficits (2)	
Losarcos	2021	5	0	0
Shields	2022	49	0 Expected	3 Expected: Weakness (2), Facial Droop + Sensory Disturbance (1)
			1 Unexpected: Mild Motor Deficit	2 Unexpected: Slurred Speech (1), Hand Weakness (1)
